# Statistics decrypted—a comprehensive review and smartphone-assisted five-step approach for good statistical practice

**DOI:** 10.1007/s00423-021-02360-0

**Published:** 2021-11-08

**Authors:** Laura C. Guglielmetti, Fabio Faber-Castell, Lukas Fink, Raphael N. Vuille-dit-Bille

**Affiliations:** 1 Department of Visceral and Thoracic Surgery, Cantonal Hospital of Winterthur, Winterthur, Switzerland; 2 Department of Neurosurgery, University Hospital Zurich, Zurich, Switzerland; 3 Department of Mathematics, Cantonal School of Wil, St. Gallen, Switzerland; 4 Department of Pediatric Surgery, University Children’s Hospital Basel, Spitalstrasse 33, 4056 Basel, Switzerland

**Keywords:** Statistics, Statistical test, Statistical analysis, Smartphone application, Five-step approach, Good statistical practice

## Abstract

**Background:**

Statistic scripts are often made by mathematicians and cryptic for clinicians or non-mathematician scientists. Nevertheless, almost all research projects necessitate the application of some statistical tests or at least an understanding thereof. The present review aims on giving an overview of the most common statistical terms and concepts. It further ensures good statistical practice by providing a five-step approach guiding the reader to the correct statistical test.

**Methods and results:**

First, different types of variables and measurements to describe a data set with means of descriptive statistics are introduced. The basic thoughts and tools of interferential statistics are presented, and different types of bias are discussed. Then in the final paragraph, the most commonly used statistical tests are described. A smartphone app accessible via QR code finally guides the reader in five steps to the correct statistical test, depending on the data used in order to avoid commonly performed mistakes.

**Conclusions:**

The five-step approach sets a new minimal standard for good statistical practice.

## Introduction

Statistic scripts are often made by mathematicians and cryptic for clinicians and or non-mathematician scientists. Nevertheless, almost all research projects necessitate the application of statistical tests. Medical practitioners and researchers are often confronted with statistical tests, be it as readers, authors, or peer reviewers of scientific publications. Misuse of statistical tests and wrongful data analyses are common and often remain unnoticed [1–7]. Reporting guidelines for main study types as e.g. presented by the equator network (https://www.equator-network.org) intend to improve health research literature by using stringent reporting recommendations. As many scientific journals adapted these guidelines as a minimal standard, there is currently no readily available guide to tackle the problems of incorrect use of statistical tests. The aim of the present article is to support scientists in understanding the most frequently used statistical expressions and to perform the most commonly used statistical tests independently and correct. The accompanying smartphone app furthermore intends to avoid commonly performed statistical mistakes in health-related research by guiding the reader in five short steps to the correct statistical test depending on the data used.

1. **Five-step approach for statistical analysis**

Attempting to tackle the problems of inadequate statistical analyses in research, a five-step procedure was created consisting of five key questions that will lead the researcher to the correct statistical test:

I) What kind of data do you have?
  - Qualitative data
  - Quantitative data
II) Do you have different observations of one single group of patients or is each observation from a different patient?
  - Paired observations
  - Non-paired observations
III) Is your data normally distributed?
  - Normal data: parametric tests
  - Non-normal data: non-parametric tests
IV) Do you want to know if groups are generally different or specifically if one group is bigger/taller/higher than the other(s) and not the other way around?
  - One-tailed *p* value of a specific test
  - Two-tailed *p* value of a specific test
V) How many groups do you compare: 2 or more?
  - Tests for two groups
  - Tests for more than two groups
    Find the according steps in the application, freely accessible through the following QR code:

## Variables

A variable relates to everything that is measured in a study. A dependent variable represents the outcome or effect that is measured during an experiment, whereas the independent variable describes its cause or input. If an investigator, e.g., examines the effect of smoking on lung cancer incidence, “smoking” reflects the independent and “lung cancer” the dependent variable. More importantly variables are categorized into qualitative and quantitative variables.

### Qualitative variables

Qualitative variables can be categorized in either two (= binary, e.g., gender: male and female) or more categories (= nominal, where the variables nominate a condition, e.g., blood types). [8] Important to know: It is not allowed to calculate nominal variables; neither if numbers are assigned to nominal variables (e.g., male = 0, female = 1). Ordinal variables are a special subgroup of nominal variables where the groups can be arranged in a logic order. Often applied examples of ordinal variables are the stage of cancer or pain scores such as the visual analogue scale (VAS). There is a natural order: A VAS of 10 is worse than a 9. And even though a pain rating between zero and ten inveigles to say that a pain reduction from ten to eight is equal to a pain reduction from five to three, this might be incorrect as we cannot assume that all patients confronted with the VAS interpret each unit of increment similar to the previous or the following one. A tumor stage 3 is worse than a tumor stage 1 of course, but one cannot say how much worse.

### Quantitative variables

Quantitative variables describe measurable factors (such as age and height) that might be calculated similar to numbers in mathematics, which is why they are also called numerical. Quantitative variables are typically associated with units (e.g., years, cm).

### Paired versus non-paired observations

After defining the type of variable, the researcher needs to define whether his observation is paired or unpaired. Paired observations examine the same variable at two different conditions or measured repeatedly at different times. For example, the patient’s weight (quantitative variable) is measured before and after a specific diet; this would be a paired set of variables (pre- and post-intervention). More commonly, data are non-paired, where different independent groups are compared [9]. If we compare the weight of soccer players versus basketball players, we compare unpaired data. Roughly, paired observations arise from the same individual, whereas unpaired observations arise from different individuals. Important exceptions are natural pairs such as twins or—depending on the outcome—siblings, couples, or matched cohorts.

### Parametric versus non-parametric statistical procedures

To finally analyze the data, the correct statistical test needs to be chosen. Data distribution is therefore of great relevance (see Distribution of data): Parametric statistical procedures rely on assumptions about the shape of the distribution (i.e., assume a normal distribution) [10]. Non-parametric test rely on no or few assumptions about the shape or parameters of the population distribution [11].

Non-parametric tests are less powerful than their parametric counterparts. To assess if data is (likely) normally distributed, different tests for normality (including the Kolmogorov–Smirnov test or the Shapiro–Wilk test) may be applied [12, 13]. More important, there are some situations where it is evident that non-parametric tests MUST be applied. These include data with outliers/extreme values, imprecise data, when the outcome has clear limits of detection (e.g,. a scale measuring the weight only up to 150 kg), or skewed data (discordance of mean and median (see descriptive statistics)). In cases where ordinal data, such as the numeric rating scale (NRS) or ranked outcomes, are analyzed, the evidence is not that clear: Especially scores with many values, such as the Karnofsky performance status scale [14, 15] (ranging from 0 to 100 percent) are often analyzed using parametric methods. With increasing sample size, the assumption of normality gets less important for basic parametric tests, such as the t-test, and these analyses deliver valid results. [11]

### Descriptive statistics

Descriptive statistics are used to describe data with text, using tables and/or figures. The objective of descriptive statistics is to integrate and communicate the data to the reader without interpreting it. So the descriptive statistic describes our own data set, not the general population.

### Median, mean, and mode

The median describes the 50% percentile, meaning that 50% of values are above and 50% are below to the median [16]. The median weight of five different patients weighting 50 kg, 60 kg, 70 kg, 100 kg, and 110 kg is 70 kg. Interestingly, if the two heaviest patients are exchanged, the median weight of five patients weighting 50 kg, 60 kg, 70 kg, 200 kg, and 300 kg remains 70 kg. Hence, the median is stable against outliers. Even if the heaviest patient was 3000 kg, the median remained 70 kg. Of note, the median may be used for ordinal (tumor stage, pain score, etc.) and for non-normally distributed quantitative (age, height, etc.) data. But again, a median VAS of 3 does not give us as much information as the count and percentage of each category.

**Fig. 1:**
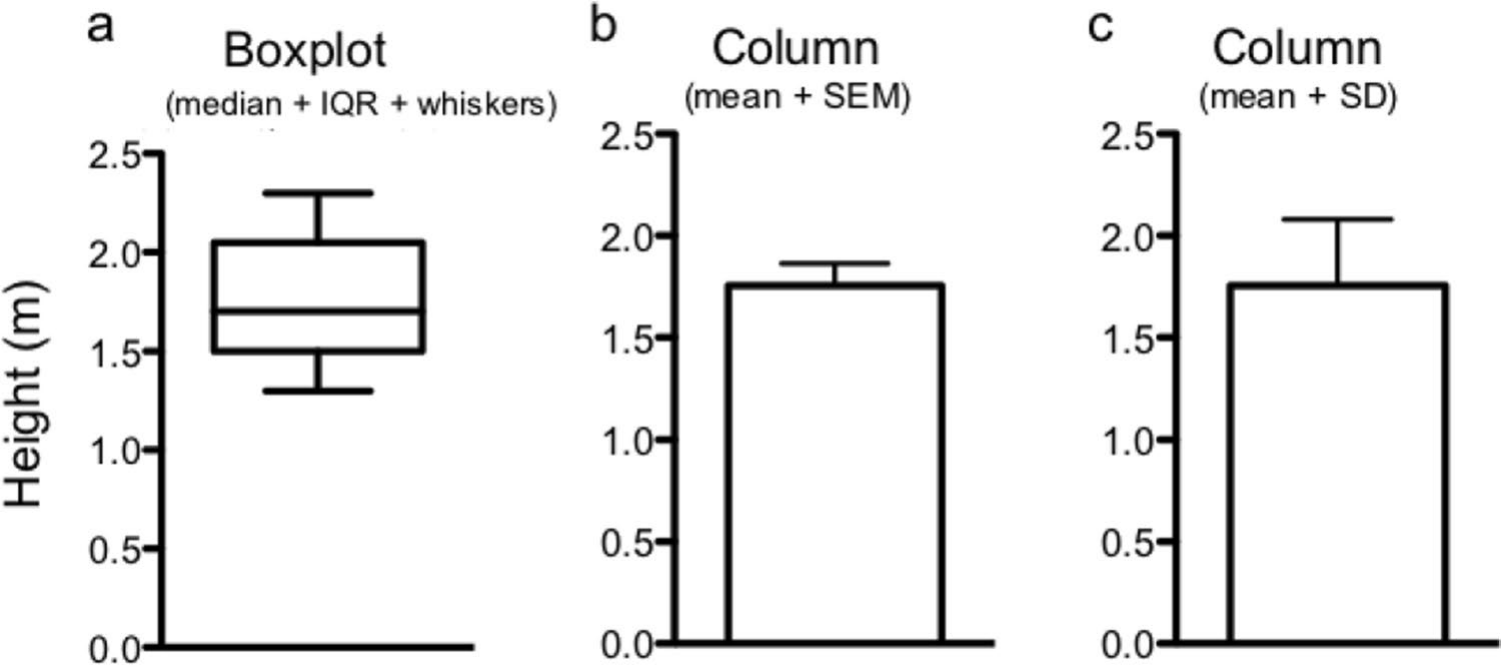
Box plot and columns displaying the height in a cohort of *n* = 9 people. Boxplot representation (**a**): The line within the box represents the median, while the upper and lower part of the box display the 75th and the 25th percentile. The meaning of the whiskers has to be defined by the authors and may represent maximum and minimum values (example here), 5th and 95th percentile, or other values. The same data is represented as columns showing mean and SD (**b**). **c** Age according to body height in a scatterplot

The mean describes the average and is easily calculated by adding all measured values dividing by the total number of values [17]. Using our experiment above, the mean of the first cohort of patients is (50 kg + 60 kg + 70 kg + 100 kg + 110 kg) = 390 kg / 5 = 78 kg. Exchanging the heaviest two patients again (as above), the mean would change to (50 kg + 60 kg + 70 kg + 200 kg + 300 kg) = 680 kg / 5 = 136 kg. The mean is much less robust to outliers than the median. For normally distributed data (i.e., 50 kg, 60 kg, 70 kg, 80 kg, 90 kg) mean and median are the same (70 kg). If the data is skewed (i.e., asymmetrically distributed around its mean), then mean and median differ from each other (see Distribution of data). This simple assessment can be used to get a first impression of our data and may already imply for which data non-parametric tests might be needed (Fig. 1). Finally, the mode is the most frequently occurring value within a set of numbers.

### Range, interquartile range, variance, and standard deviation

One way to describe the distribution of data is the *range* that describes the difference between the highest and the lowest value (i.e., the difference between the 100th and the 0th percentile). The range may be used for ordinal and for quantitative variables.

Often, researchers use the interquartile range (IQR), i.e., the difference between the 75th and the 25th percentile to describe distribution of data. One possibility to display percentiles (i.e., 25th percentile, median, and 75th percentile) are box plots that are commonly used by clinicians (Fig. 1).

The distribution of data (around the mean) can be given as standard deviation (SD) or standard error of the mean (SEM), with the latter being calculated by dividing the SD by the square root of the number of values (n) (SEM = SD / √n) [17–19]. For normally distributed data, 68% of values lie within one SD, and 95.5% lie within two SDs from the mean. In general, quantitative variables which are normally distributed are reported as mean + SD and non-normal quantitative data are reported as median + range or median + IQR.

Qualitative variables on the other hand are typically reported as count and percentages.

Mean, median, and mode are considered measurements of the central tendency of a data set, while variance, standard deviation, range, and interquartile range are called measurements of dispersion.

### Distribution of data

**Fig. 2:**
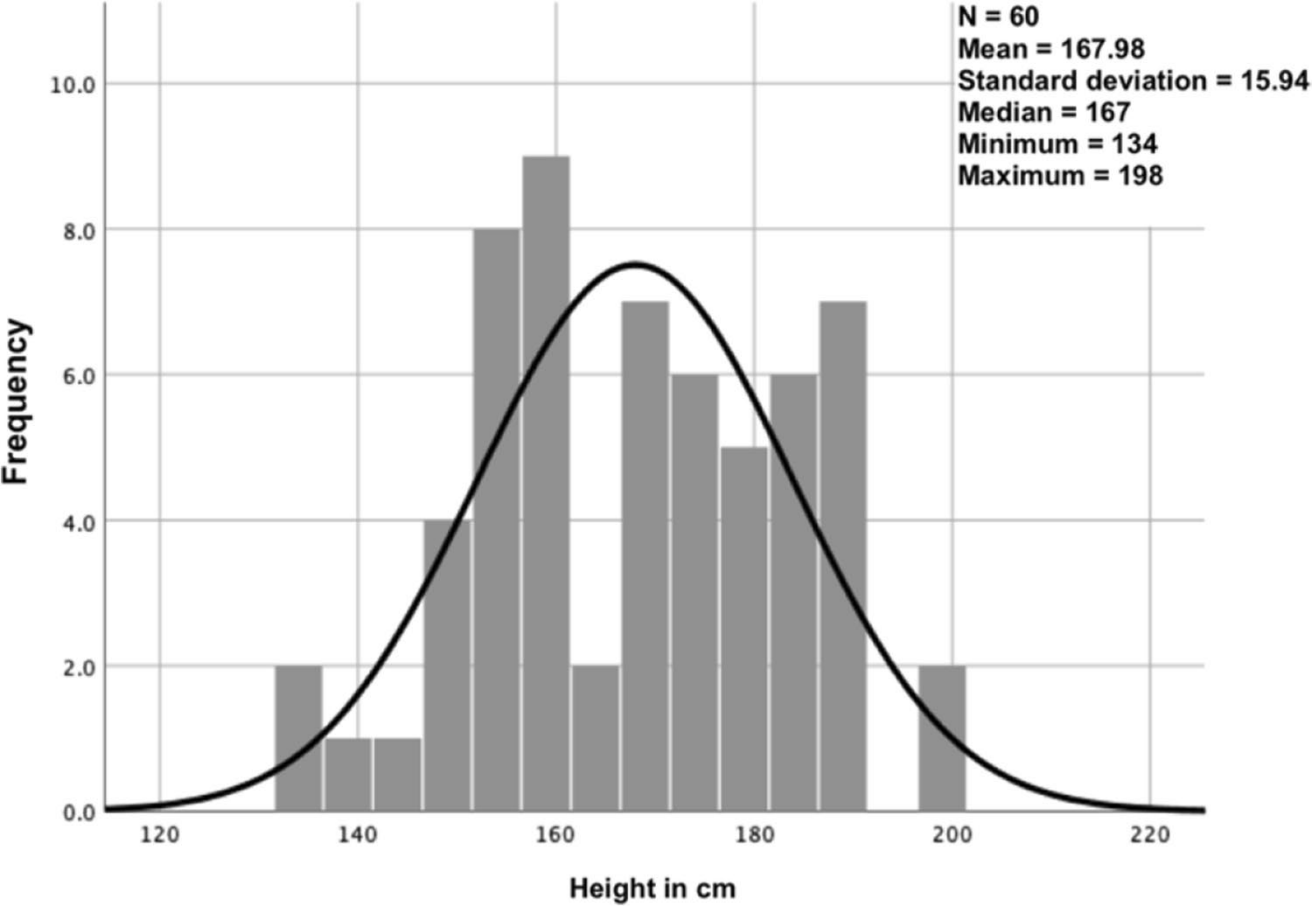
Frequency distribution of the heights of *n* = 60 patients. The black overlying curve represents the standard normal distribution and helps us to visually assess for normality. In our case, we see how difficult the visual assessment can be

One possibility to represent the distribution of data is a graph showing all possible values (or intervals) of the data (*x*-axis) and how often they occur (*y*-axis) (Fig. 2). The most well-known data distribution is the normal or Gaussian distribution, where the data builds a bell-shaped curve. We are often confronted with limited sample sizes, and therefore the assumption of normality is not satisfied. A simple indicator of a non-normal distribution is a large disconcordance of the mean and the median of a population. In cases where the same variable was used in a previous, larger study (e.g., age of patients undergoing laparoscopic appendectomy in a cohort of 2000 patients) and we know it was normally distributed, we may assume a normal distribution in our data set as well (only 20 patients undergoing laparoscopic appendectomy), even if it is significantly smaller.

### Relative risk and odds ratio

Often confusing, but easily understandable are the relative risk and the odds ratio. Taking a normal dice, the risk of throwing a “6” is 1/6 = 16.6% (i.e., the number of positive events (6) divided by all possible events (1, 2, 3, 4, 5, 6)), whereas the odds of dicing a “6” is 1/(6–1) = 1/5 = 20% (i.e., the number of positive events (6) divided by all negative events (1, 2, 3, 4, 5)). If we take two populations (e.g., 100 smokers vs. 100 non-smokers) and we intend to calculate the incidence of lung cancer, both, the relative risk and the odds ratio may be applied. If 20 smokers and 5 non-smokers sicken from lung cancer, the risk for smokers to sicken is 20/100 = 20%, and the risk for non-smokers to sicken is 5/100 = 5%. Hence, the relative risk is 20/100 divided by 5/100, which is 0.2/0.05 = 4, meaning that the risk to sicken from lung cancer is 4 times higher in smokers than in non-smokers. Applying the same example, the odds to sicken for smokers is 20/(100–20) = 20/80 = 0.25, and the odds to sicken for non-smokers is 5/(100–5) = 5/95 = 0.052. Hence, the odds ratio is 0.25/0.052 = 4.75. Odds ratios are typically reported with the corresponding 95% confidence intervals.

### Correlation

The concept of correlation describes the relationship between two variables. When one increases, we want to know whether the other one increases or decreases as well. The correlation coefficient (*r*) ranges from − 1 to 1 indicating there is a strength (a value between 0 and 1) and a direction (- or +) of the correlation. In case of perfect linear correlation, the direction and the quantity of the change are similar; both variables move with the same slope into the same direction (correlation coefficient equals 1). In case one variable behaves exactly opposite to the other, we have a negative correlation (with a maximum of − 1 as correlation coefficient). Depending on the distribution of our data, two main types of correlation analysis are used [20]: Pearson correlation which can be applied on normally distributed data to describe a linear correlation in continuous data. Linear correlation means that an increase in one variable results in a proportional change (increase or decrease) of the other variable. And Spearman rank-order correlation which compares ranks (and not the actual numbers) is applied in case of non-normal data and detects monotonic correlation in continuous or ordinal data. Monotonic correlation describes the change of one variable leading to a change in the other variable but not always to the same extent; the direction remains similar, but the proportion of change may vary. The translation from a correlation coefficient into words depends on the application and the research field. For further guidance, we recommend the following publications by Evans [21] and Akoglu [22].

A fundamental aspect of correlation is that it has nothing to do with causation. Correlation describes a dependence of two variables not an actual causation. A famous and very memorable example is the correlation of stork populations and human birth rates across Europe [23]. There is a statistically significant linear correlation, but as we know, there is no causality. One changes with the other, but not necessarily because of the other. Another important aspect is that with Spearman correlation only monotonic and with and Pearson correlation only linear correlation are measured but other non-linear, very strong relationships may exist even if both correlation coefficients are 0.

### Inferential statistics

If we want to know how many people in the world have brown eyes, we are unlikely to actually meet each person and note the eye color. What we can do is to look at a subset of people (e.g., everyone in the same room), count the brown-eyed subgroup within this sample, and estimate the prevalence of brown eyes. The objective of inferential statistics is to infer the likelihood that the observed results can be generalized to other samples of individuals/to the general population [18]. The aim of a statistical analysis is usually to examine a set of data from a sample population and to extrapolate the findings to the complete population. Therefore, the analyzed sample needs to be representative of the population. But as we do not know the complete population, we can only estimate how generalizable the results found in the sample are for the population.

### Confidence interval

Generally, the 95% confidence interval is used in clinical (and other) studies [24]. The 95% confidence interval of the sample mean for five game scores 86, 95, 99, 106, and 120 ranges from 85 to 117, meaning that the interval from 85 to 117 includes the (unknown) population mean with a probability of 95%. Which means that the 95% CI of the sample mean game score states that if we repeat the complete experiment 100 times (selection of 5 individuals, observing their game score), 95 times the CI will overlie the population mean game score. And, 5 times it will not!

To calculate the 95% CI of a normally distributed sample mean, we use the standard error of the sample. The mean of this sample follows the normal distribution, and the standard error is a measure of dispersion of the normal distribution. For normally distributed data, 95% lies within the sample mean ± 1.96 dispersion (standard error). So, in case of a normal distribution, the sample mean ± 1.96 standard error reflects the 95% CI for the sample mean.

### Null hypothesis and alternative hypothesis

H0 states that there is no relationship between two measured phenomena (e.g., smoking and lung cancer). Results are obtained by chance alone. The alternative hypothesis (H1) is the rival hypothesis (that there is a relationship). The H0 and H1 are mutually exclusive. They cover all possibilities. In statistics, the researcher either rejects the H0 (In this case H1 is found to be true) or fails to reject the H0 (in case the H1 cannot be proven to be correct). The H0 cannot be proven. Our analyses focus on H1, which can be proven or fails to be proven.

### Type I and type II errors

Since we can only approximate the truth with statistics, there is always a chance of error. A type I error is a false-positive finding, e.g., a positive pregnancy test in a man. It is the rejection of a true null hypothesis (i.e., one assumes a relationship in case there is no relationship), also called a rejection error. The probability of a type I error is described with the significance level alpha (α). The significance level has to be determined prior to the analysis of our data. Typically, a significance level of 0.05 is chosen. This means that if we repeat our hypothesis test 100 times and alpha is set at 0.05, we would falsely reject a true H0 in 5 cases or 5% of the times we repeated the hypothesis testing. Hence, choosing an alpha of 0.05, we tolerate false-positive findings (e.g., pregnancy tests in men) in 5% of cases.

A type II error is a false-negative result, e.g., a negative pregnancy test in a pregnant woman. The probability of a type II error is denoted beta (β) or acceptance failure. If beta is set at e.g. 0.2, we would tolerate false-negative results (negative pregnancy tests in pregnant women) in 20% of cases.

In summary, whereas type I errors describe false findings, type II errors describe misses.

### p Value

The *p* value (or probability value) tells us how well our data fits to the null hypothesis (0, not fit at all; 1, fits very well). It reflects the probability to get the current (or even a more extreme) result, given that the null hypothesis is true. As a probability, the *p* value ranges between 0 and 1. A low *p* value indicates strong evidence against the null hypothesis. If the *p* value is less than (or equal to) alpha (0.05, 0.01, or 0.001, etc.), then the null hypothesis is rejected. The alternative hypothesis is then accepted [24].

## Statistical power

We learned that beta (β) is the probability to make a type II error (= acceptance error; false-negative result). Statistical power of a hypothesis test is defined as 1-β, the probability to reject a false H0 (true negative result), which is in fact the probability of what we would like to achieve with our hypothesis test. In other words, 1-β is the probability of NOT committing a type II error [24, 25].

### Sample size calculation

Intuitively, we know that the more eyes we are able check for color, or the more IQs we measure (= the bigger our sample from the world population), the better our estimation of the truth about the prevalence of brown eyes or the mean IQ within the world population will be. This is one of the fundamentals in power and sample size calculation: This also explains that we sometimes find a statistically significant difference which has no clinical significance (e.g., a difference of one IQ point between two groups can be statistically significant but will not influence any clinical decisions).

**Fig. 3:**
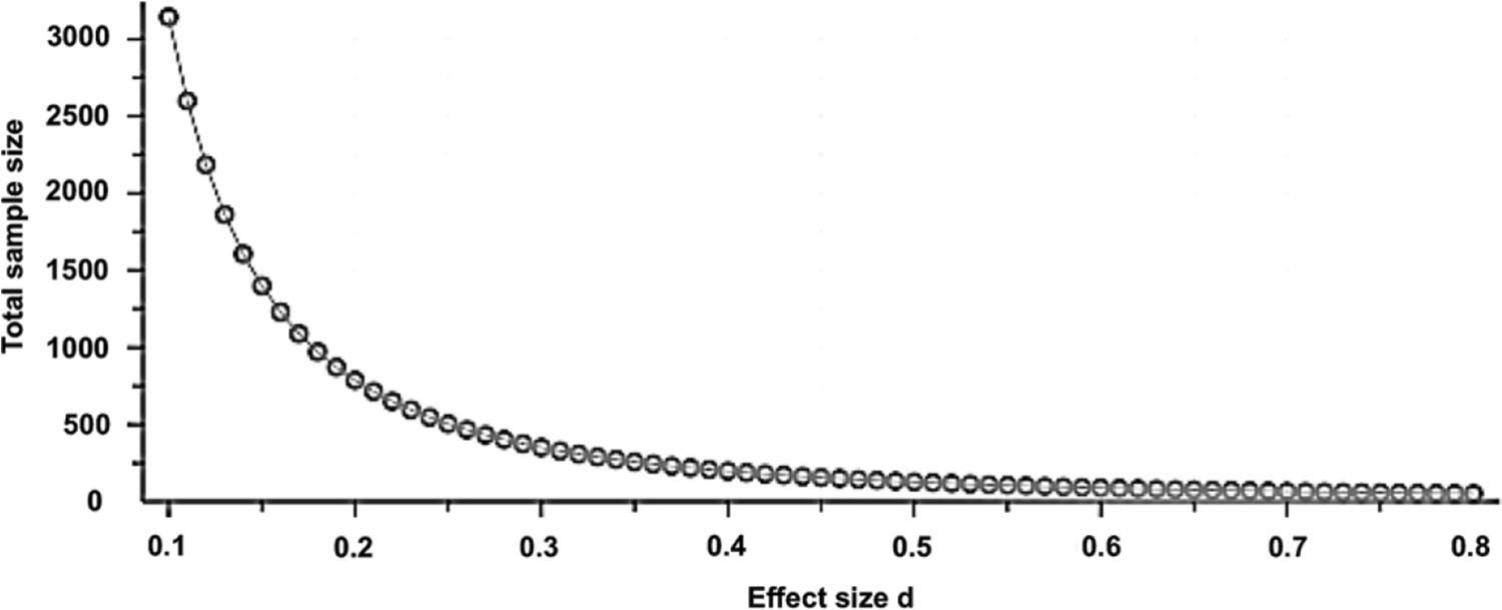
Sample size calculation: Significance level alpha is set at 0.05. Power is set at 0.8. The effect size (calculated from expected difference between groups and standard deviation) is given on the *x*-axis, and the calculated sample size is given on the *y*-axis. Decreasing the effect size drastically increases the sample size needed for the experiment

Usually, we want to know how many individuals have to be included in a study to get a certain power in detection of a difference at a predefined significance level. There are several tools for sample size calculations with the G*Power calculation program (Version 3.1.9.3, http://www.gpower.hhu.de/) being the one used by the present authors. Other freely available online tools are https://www.statmethods.net/stats/power.html, https://www.stat.ubc.ca/~rollin/stats/ssize/, https://clincalc.com/stats/samplesize.aspx, https://www.gigacalculator.com/calculators/power-sample-size-calculator.php, and https://select-statistics.co.uk/calculators/sample-size-calculator-two-means/, just to name a few. To perform a sample size calculation, 5 points need to be defined in advance: the significance level alpha, the power, the expected difference between the two compared groups, the expected standard deviation, and the statistical test. Whereas the significance level alpha is typically set at 0.05, and the power at (no less then) 80%, we need to anticipate the latter two measures (from literature review or previous experiments) and then calculate the effect size (d). Let us say we plan to compare two operation methods, and our primary outcome is the duration of surgery, which means that we calculate the power for a *t*-test which we will apply to compare mean surgery time between the two groups. We know from the literature review or from pilot studies that mean operation time of operation method A is around 50 min, whereas mean operation method B takes about 35 min. Standard deviation is 18 min. The effect size is calculated as difference of the means (50 min – 35 min = 15 min) divided by the standard deviation (15 min / 18 min = 0.83). Effect sizes of 0.2, 0.5, and 0.8 correspond to small, moderate, and large effects, respectively. Figure 3 should help us to better understand the importance of the effect size on the calculated sample size.

When sample size calculation is conducted the same five parameters should be reported: the significance level alpha (usually 0.05), the power (usually ≥ 80%), the expected difference between the two compared groups, the expected standard deviation, and the statistical test. For the latter two, the source of information should be reported in order to make the power calculation replicable.

Possible consequences of an underpowered study and their impact on the false discovery rate have been widely discussed in the literature [26–28].

Hoenig and Heisey and Zhang and colleagues wrote interesting articles on post hoc power analysis, which describes the calculation of statistical power after data collection and analysis of the data (post hoc = made or happening after an event, neither planned nor decided a priori) [29] [30]. However, in general, post hoc power analyses cannot be recommended and remain the subject of extensive debates. We refer the interested reader to the following references [29–33].

### Bias

Bias is the tendency to over- or underestimate a parameter. It describes (confounding) factors that falsify an interpretation of an experiment [34]. For researchers, it is important to realize that bias can occur at any stage of a research project: during research planning (e.g., selection bias), during data analysis (e.g., detection bias), and during research reporting (e.g., reporting or publication bias) [35, 36]. Furthermore, researchers should be aware how they can avoid bias [37, 38]. We summarized the most important types of bias with examples and possible measures for their prevention in Table 1.

**Table 1:** Types of bias

Type of bias	Description	Example	Prevention
Selection bias	Some participants are more likely to be selected for a study. Included participants are not representative of the population	Unemployed people more likely to participate in a time-consuming study	Allocation concealment, sequence generation
Detection bias	A certain condition is more likely to be detected in a subgroup of participants due to systematic differences in how outcomes are determined	Detection of appendicitis by ultrasound in thin versus obese patients	Blinding of outcome assessment
Reporting bias	Positive results and correlations are more likely to be reported	Non-finding or negative finding is not published	Preemptive determination of outcomes of interest
Exclusion bias	A certain population is more likely to be excluded from a study	Pregnancy, vulnerable patients such as small children or elderly are not included	Preemptive definition of exclusion criteria and consideration of these during discussion of the results found
Attrition bias	Loss of follow-up of a certain subgroup of participants	Elderly people not reachable via email	Reporting of incomplete outcome data, intention to treat analysis
Performance bias	Systematic differences between the groups regarding the exposure or care other than the intervention	Group receiving a drug gets more frequent blood examinations	Double blinding

Adapted from [38]

### The most common statistical tests

Statistical mistakes in health-related research are common (probably more common than expected) and often go undetected. These errors e.g. consist of using multiple *t*-tests for multiple group comparisons, using paired tests for unpaired data (and vice versa), using a *t*-test under non-parametric conditions, etc. and hence base on misunderstanding and/or neglecting of basic statistical concepts [3]. As good statistical practice guidelines and recommendations in health-related research are currently lacking, a five-step approach to the correct statistical test (depending on the used data) was created by the presenting authors that can be freely used as smartphone app by scanning the QR code attached. Depending on the type of data (i.e., parametric vs. non-parametric, paired vs. unpaired, etc.) are hereby assessed by five specific questions leading the reader to the correct statistical analysis. As mentioned above, the first step includes the type of data we have. When we look at a typical surgical publication, we have typical outcome data such as length of stay at the hospital, numeric rating scale for postoperative pain levels or duration of a procedure (quantitative data), and binary data such as did the patient have a complication, was a chest tube inserted, and ordinal data (e.g., Clavien-Dindo classification of postoperative complications [39]). Depending on the data type, different statistical tests are applied.

### Qualitative data analysis: crosstables and Chi-squared test

For comparison of proportions of a qualitative variable, a simple crosstable (2 × 2 table) with a Chi-squared test can be used (Table 2). A crosstable typically includes 2 or more groups and the according proportions of a variable of interest per group. Comparing e.g. the proportion of females in two groups (A and B), the H0 is that no difference between group A and B exists. H0 = proportion of females in group A = proportion of females in group B. The alternative hypothesis is that there is a different proportion of females in group A compared to B. This hypothesis can be tested with a *Chi-squared test*.

**Table 2:** Example of a crosstable (2 × 2)

N (%)	Group A	Group B
Males	2 (20%)	4 (40%)
Females	8 (80%)	6 (60%)
Total	10 (100%)	10 (100%)

The proportion of males and females within two groups of a sample population are compared

In cases of small sample sizes (in a 2 × 2 table, if one of the fields expected value is < 5; in larger tables, if > 20% of the fields have an expected value < 5), a Fishers exact test should be used [8]. The Fishers exact test is less powerful, but it does not require a minimal number of entries for each field. Some statistical programs automatically recommend the Fishers exact test for a certain sample size (e.g., SPSS, IBM corp., Armonk, NY) [40–42].

#### Quantitative data and ordinal data analysis

**Fig. 4:**
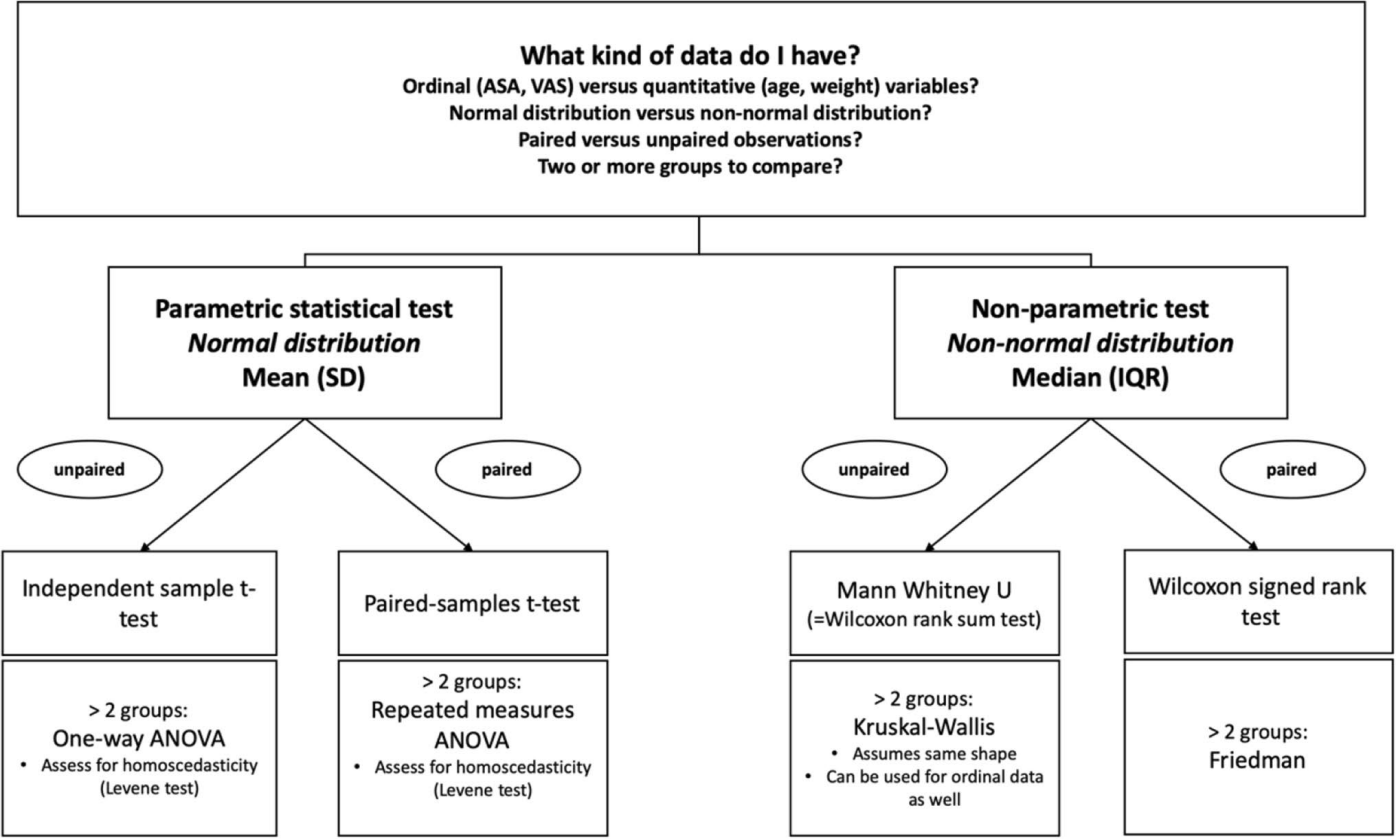
Decision-making when a statistical test is applied

Figure 4 summarizes the most common statistical test and when they can or cannot be used.

#### t-Test

**Fig. 5:**
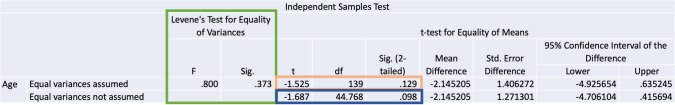
SPSS output for the comparison of the mean age between two groups. Framed in green, we see the Levene test for equality of variances, which SPSS automatically applies when we compare means with a *t*-test. A significant *p* Value for the Levene test means that homogeneity of variance cannot be assumed. Therefore, the second line from the SPSS output (framed in dark blue) “equal variances not assumed” contains the *p* value from the correct *t*-test (Welch’s *t*-test). In Fig. 5, the *p* value for the Levene Test is > 0.05, which means that homogeneity of the variance can be assumed: The upper line, framed in orange, contains the correct *t*-test (Student’s *t-*test); the *p* value we are looking for is *p* = 0.129

For comparison of two means (and the according standard deviations) of a normally distributed,sample a Student *t*-test can be used. In cases of paired comparisons (within group), a paired *t*-test is applied; for independent comparisons (between groups), an independent *t*-test is used. A *t*-test can be one-tailed or two-tailed. Whereas in one-tailed *t*-tests, the question is only one-directional (i.e., is group B bigger/faster/larger than group A?), it is two-directional in two-tailed *t*-tests (i.e., is there a difference between groups A and B, e.g. bigger or smaller?). If we perform a one-tailed *t*-test, we will not be able to say if group A is smaller/slower/shorter than group B. Most of the time, a two-tailed *t*-test should be used. As Kwak and colleagues pointed out, the assumption of normality becomes less important with the increasing sample size for the *t*-test [43]. Generally, the assumption of equal variances needs to be fulfilled for a Student *t*-test; if not, the Welch’s *t*-test should be applied. A commonly used statistical software, such as SPSS (IBM corp., Armonk, NY), automatically delivers both results (Student *t*-test and Welch’s *t*-test along with the analysis of variance); the user simply needs to read the output correctly (Fig. 5).

#### ANOVA

The analysis of variance is helpful in cases where we want to examine the means of more than two independent groups.

One-way ANOVA can be used for comparison of the means of more than two independent groups [9].

Each level of the independent variable needs to be approximately normal distributed and homoscedastic. Homoscedasticity in one-way ANOVA is the assumption of homogeneity of variance, and most statistical software (e.g. SPSS) automatically assesses for homoscedasticity when a one-way ANOVA is performed. Homoscedasticity can be assessed with a Brown-Forsythe test or the more popular Levene test.

Repeated measures ANOVA can be used to compare the means of more than two dependent groups. It is the equivalent of a paired *t*-test for more than two groups. The assumptions are similar as for the one-way ANOVA: approximately normal distributed variables and homoscedasticity.

When we use an ANOVA to compare a variable between more than two groups, we will get a single *p* value, stating that there is, or is not, a statistically significant difference somewhere between the groups we compare. But we will not yet know which groups are significantly different from each other. For example, when we analyze the preoperative stress levels of a patient depending on his mother tongue (English, Spanish, German, Italian or French). The analysis of variance might tell us that there is a significant difference somewhere within our data (the result of the ANOVA will be a single *p* value below 0.05), but we will not know which two groups are statistically significant different from each other: We have to compare group by group (e.g., English versus Spanish, English versus German, English versus Italian, English versus French, Spanish versus German, Spanish versus Italian, Spanish versus French, German versus Italian, German versus French, and Italian versus French). This is done with a post hoc analysis, which means that we “re-examine” the same data to detect which groups are statistically significant different from each other. Again, software such as SPSS has automated tools for post hoc analyses after ANOVA; we advise the interested reader to consult the IBM homepage for further guidance [44].

#### Mann Whitney U test

Mann Whitney *U* test is also called the Wilcoxon rank sum test [45] which is used for comparison of non-normally distributed data between two independent groups [8].

It is the non-parametric equivalent of an independent *t*-test. It is not just a simple comparison of two medians; it is a test of location and shape. That is why it is possible that you find statistically significant differences between two groups with a (numerically) identical median. There is one important assumption that should be fulfilled when a Mann Whitney *U* test is used to compare medians: the assumption of same shapes of the distributions of the different groups.

#### Kruskal–Wallis test

If we want to compare more than two groups and the dependent variable is ordinal or the dependent variable is continuous but the assumptions for an ANOVA are not fulfilled, we can use a Kruskal–Wallis test. It is the non-parametric equivalent of a one-way ANOVA. It is an extension of the Wilcoxon rank sum test or Mann Whitney *U* test to examine more than two groups.

So this test is used for comparison of non-normally distributed, independent data of more than two groups.

Again, the assumption of same shapes of the distributions of the different groups should be fulfilled. In cases where we cannot use a one-way ANOVA due to heteroscedasticity, the assumption of same shapes is not fulfilled either; the Kruskal Wallis is not a good option. In these cases, a Welch’s ANOVA can be used instead if your data is approximately normal distributed. In cases with heteroscedasticity and non-normally distributed data, there is no simple answer on which test should be performed; there are however some very useful publications on this matter [46, 47].

#### Wilcoxon signed-rank test

It can be used for comparison of two non-normally distributed, paired measurements (e.g., median of a preoperatively measured characteristic compared to the median of the same characteristic measured postoperatively).

The Wilcoxon signed-rank test ranks the differences between the samples of interest. It includes the magnitude of the difference and the sign (positive or negative difference). An alternative is the sign test, which does not take into account the magnitude of the differences, only the signs. It is therefore less powerful and not used very often.

#### Friedman test

If more than two groups of paired data that are not normally distributed are compared, a Friedman test should be used. An example would be the patient’s quality of life (with a score from 1 to 100) at three different points in time.

### Multiple testing: the Bonferroni correction

Researchers may have an outcome at several time points and hence perform multiple tests. To consider the problem of multiple comparisons, certain corrections are performed. Different correction methods tests have been described [10] with the Bonferroni correction being the most well-known: hereby, to achieve a global alpha-level of 5%, each individual hypothesis is tested at α = 0.05/*x*, with *x* = the number of comparisons performed during the experiment [48]. The problem with multiple testing is that with each test we run, the probability of a type I error (false-positive finding) increases. If we run 100 tests, the likelihood that at least one test will show a ‘significant’ (p < 0.05) difference between groups just by chance will be extremely high. To overcome this problem, a correction is applied. If we compare pain scores (numeric rating scale, NRS) at different time points post-surgery with preoperative NRS, we repeat the same statistical test and must take the repeated measurements into account. Hence, if we compare 10 postoperative NRS from different timeslots to the preoperative NRS of the same patient, we will have to divide alpha (0.05) by 10 (0.05/10 = 0.005). Only *p* values of 0.005 or less would then be considered statistically significant differences. A clear disadvantage of the Bonferroni correction (and likely the reason why it is not performed routinely) is its stringency. By using the Bonferroni correction, we not only decrease the number of false positive but also of true positive findings. Hence, the Bonferroni correction is often considered too conservative. A less strict (but technically more difficult) correction is the Holm-Bonferroni method. Hereby, the comparisons with *p* values of < 0.05 are grouped with the lowest value being the first and the highest being the last comparison. The significance level alpha is decreasing from each comparison to the other (alpha/n, alpha/(n-1), alpha/(n-2)). Whenever a *p* value is higher than alpha, no further comparisons can be made. Applying our example above again, let us say we find that NRS on postoperative day 1 (POD 1) compared to preoperative NRS are significantly higher (*p* = 0.0001), also on POD 5 compared to preoperative NRS values (*p* = 0.0003), and still higher on POD 10 (*p* = 0.03), and finally, lower on POD 30 (*p* = 0.04). Our first *p* value (0.0001) is lower than alpha/10 (0.005) and hence considered significant. Our second *p* value (0.0003) is lower than alpha/(10–1) = alpha/9 = 0.0056 and likewise considered significant. Our third *p* value (0.03) is higher than alpha/(10–2) = alpha/8 = 0.0063. It is hence not a significant finding. Furthermore, we have to stop here and are not allowed to do any further comparisons.

The five-step approach for statistical analysis can be accessed as smartphone application by scanning the following QR code:

## Limitations

The five-step approach includes some of the most basic and commonly applied statistical tests, and this manuscript aims to explain some of the basic concepts of statistical analysis. However, the application only covers common tests used in surgical publications, which is in fact only a small sample within a multitude of statistical concepts and tests. This manuscript cannot replace a book on statistical methods or the help of a statistician with a complex data set.

## Further development of the application

As we included a contact form in the app, we hope to collect some real-life examples of statistical problems which might help and guide other clinicians when analyzing their own data. We plan a frequently asked question section where we publish (with the authors consent) some of the most frequent and important questions we receive.

The use of the application is meant to be freely available; we do not plan to commercialize the app, as we also do not offer professional statistical support but rather an exchange of thoughts and perspectives among colleagues.

## Conclusion

We hereby present an easily accessible and straight forward method for basic statistical analyses. The application enables any non-mathematician to decide what statistical test might be applied to the data and especially what test should not be used and why. The five-step approach sets a new minimal standard for good statistical practice.
